# Mn^2+^-Mediated Antiviral Activity Through Both the cGAS-STING-IFN and ROS-Apoptosis Pathways in Porcine Alveolar Macrophage Cells

**DOI:** 10.3390/vetsci13040396

**Published:** 2026-04-17

**Authors:** Wanglong Zheng, Yajing Chang, Anjing Liu, Chenyang Zhang, Weilin Hao, Tianna Chen, Qing Lu, Zhiyu Wang, Wei Wang, Nanhua Chen, Jianzhong Zhu

**Affiliations:** 1 College of Veterinary Medicine, Yangzhou University, Yangzhou 225009, China; wanglongzheng@yzu.edu.cn (W.Z.); mx120241045@stu.yzu.edu.cn (Y.C.); zalm1314mx120220988@stu.yzu.edu.cn (A.L.); 222204147@stu.yzu.edu.cn (C.Z.); mz120231722@stu.yzu.edu.cn (W.H.); 232002102@stu.yzu.edu.cn (T.C.); 232002113@stu.yzu.edu.cn (Q.L.); mx120251006@stu.yzu.edu.cn (Z.W.); mx120251005@stu.yzu.edu.cn (W.W.); hnchen@yzu.edu.cn (N.C.); 2 Comparative Medicine Research Institute, Yangzhou University, Yangzhou 225009, China; 3 Joint International Research Laboratory of Agriculture and Agri-Product Safety, Yangzhou 225009, China; 4 Jiangsu Co-Innovation Center for Prevention and Control of Important Animal Infectious Diseases and Zoonoses, Yangzhou University, Yangzhou 225009, China

**Keywords:** manganese ion (Mn^2+^), cGAS-STING pathway, antiviral activity, apoptosis

## Abstract

Manganese ions (Mn^2+^) are an essential trace element within organisms spanning the entire tree of life. It has reported that Mn^2+^ exerts strong immunocompetence effects and exhibits antiviral effects against various human and animal viruses, including DNA and RNA viruses. Recently, Mn^2+^ has been found to be involved in the activation of the innate immune DNA-sensing cyclic GMP-AMP synthase (cGAS) stimulator of interferon genes (STING) pathway and subsequent antiviral function. However, the antiviral mechanism of Mn^2+^ remains unclear. In the current study, the results suggest that the cGAS-STING pathway is essential for Mn^2+^ to promote interferon (IFN) signaling, but it is not essential for triggering antiviral functions. After knocking out the STING or interferon regulatory factor 3 (IRF3) gene, Mn^2+^ still retains its antiviral activity against herpes simplex virus type 1 (HSV-1) and vesicular stomatitis virus (VSV). Furthermore, the results from transcriptomic analysis indicate that Mn^2+^ can induce a significant change in the apoptotic process in STING^−^/^−^ 3D4/21 cells. Mn^2+^ can induce cell apoptosis through the oxidative stress pathway, and inhibiting the apoptotic signal could suppress Mn^2+^-mediated antiviral activity in STING^−^/^−^ 3D4/21 cells. Additionally, dual knockout of IRF3 and caspase3, resulting in concurrent loss of IFN and apoptotic signals, eliminates the antiviral effects of Mn^2+^. In summary, the current study suggests that Mn^2+^ could exert antiviral effects not only through the cGAS-STING-IFN pathway but also via the reactive oxygen species (ROS)-apoptosis pathway.

## Simple Summary

Manganese ions (Mn^2+^) are an important trace element that can activate innate immunity and inhibit viral infection. Here, we used porcine alveolar macrophage 3D4/21 cells and gene-knockout cells to explore antiviral pathways of Mn^2+^. We found that the cGAS-STING pathway is required for Mn^2+^ to induce IFN signaling, but not essential for its antiviral activity. Even in STING-deficient cells, Mn^2+^ still suppressed HSV-1 and VSV replication. This study indicates that Mn^2+^ inhibits viruses not only through the cGAS-STING-IFN pathway, but also through the ROS-apoptosis pathway.

## 1. Introduction

Manganese ions (Mn^2+^) are an essential trace element with diverse physiological functions in biological systems. Mn^2+^ can integrate into multiple metalloenzymes, including glutamine synthetase (GS), pyruvate carboxylase and arginase, and exert essential regulatory effects on the activity of these enzymes [1]. Mn^2+^ itself is a potent natural immune stimulant that induces the production of type I interferons (IFN-I) and cytokines, thereby enhancing host antiviral immunity. Mn^2+^ markedly boosts immune responses by enhancing antigen uptake, antigen presentation, and germinal center formation. Manganese salts could function as potent adjuvants, and a colloidal manganese formulation has been developed to serve as an immune adjuvant and an efficient delivery vehicle. A colloidal manganese formulation could potently trigger both humoral and cellular immune activation when administered via intramuscular or intranasal immunization routes [2]. A study showed that mice with manganese deficiency exhibit diminished cytokine production and display heightened susceptibility to DNA viruses, which highlights the essential role of Mn^2+^ in host immune defense against such viral pathogens [3]. It was also reported that Mn^2+^ is capable of triggering immune responses against RNA virus infections, such as those caused by Newcastle disease virus (NDV), foot-and-mouth disease virus (FMDV), and Sendai virus (SeV) [4,5].

The innate immune system serves as the body’s first line of defense. It detects viruses and senses danger signals via pattern recognition receptors (PRRs) and triggers the production of interferons and proinflammatory cytokines to eliminate viral pathogens efficiently. PRRs are germline-encoded host proteins that exist either on cell membranes or within the cytoplasm [6]. Their core role is to detect microbial invasion and sterile tissue damage stimuli. PRRs mainly include Toll-like receptors (TLRs), cytosolic NOD-like receptors (NLRs), intracellular RIG1-like receptors (RLRs), and cyclic GMP-AMP synthase (cGAS) [7]. As a cytosolic DNA sensor, cGAS identifies pathogenic double-stranded DNA (dsDNA) and catalyzes the production of cyclic GMP-AMP (cGAMP), which in turn triggers the activation of STING [8,9]. Activated STING subsequently recruits TANK-binding kinase 1 (TBK1), leading to the phosphorylation of interferon regulatory factor 3 (IRF3), thereby promoting the expression of IFN-I and pro-inflammatory cytokines to enhance immune responses [9].

The cGAS-STING signaling cascade constitutes an essential innate immune pathway and exerts a pivotal function in host defensive responses. Several studies have demonstrated that Mn^2+^ could induce the production of IFN-I expression and exert antiviral effects through activating the cGASSTING signaling pathway [3,10]. Mn^2+^ are primarily stored within two major intracellular compartments including the mitochondria and the Golgi apparatus, from which Mn^2+^ can be released into the cytoplasm. It has been shown that Mn^2+^ released from intracellular organelles and manganese-binding proteins collectively elevates cytosolic manganese ion concentrations, thereby facilitating the activation of the cGAS-STING signaling pathway [3]. Furthermore, Mn^2+^ was shown to increase the sensitivity of cGAS to dsDNA and its enzymatic activity [3]. It also facilitates STING activity by boosting cGAMP-STING binding affinity. Additionally, a study revealed that Mn^2+^ activates cGAS directly, without the requirement of dsDNA as a stimulant, leading to the synthesis of 2′3′-cGAMP [10]. Structural analyses show that Mn^2+^-triggered cGAS shares overall conformational changes with its DNA-activated form, and Mn^2+^-induced cGAS uniquely forms an η1 helix, which expands the catalytic pocket [10]. This structural rearrangement facilitates substrate access and promotes cGAMP production.

Recently, an increasing number of publications have suggested that Mn^2+^ could exert antiviral effects through the cGAS-STING pathway [3,10]. However, our previous study has shown that Mn^2+^ could exert a broad antiviral function against various viruses in a cGAS-STING-independent manner [11]. The molecular mechanism of how Mn^2+^ inhibits virus replication is still unclear. In the current study, we utilized the porcine alveolar macrophage cell line (3D4/21), STING-knockout counterpart (STING^−/−^3D4/21), IRF3-knockout 3D4/21 (IRF3^−/−^3D4/21) cells, and dual-knockout IRF3 and caspase3 3D4/21 (IRF3^−/−^STING^−/−^3D4/21) cells as models to investigate the antiviral mechanisms of Mn^2+^ independent of the cGAS-STING pathway.

## 2. Materials and Methods

### 2.1. Cells and Viruses

Porcine alveolar macrophage (3D4/21) cells were purchased from ATCC (American Type Culture Collection). STING^−/−^3D4/21, IRF3^−/−^3D4/21, and IRF3^−/−^STING^−/−^3D4/21 cells were constructed and stored in our lab. The 3D4/21 cell line and its corresponding knockout derivatives were cultured in RPMI 1640 medium (Hyclone Laboratories, Logan, UT, USA) supplemented with 10% fetal bovine serum (FBS) and 1% penicillin–streptomycin mixture. Cell maintenance was performed at 37 °C in a humidified incubator supplemented with 5% CO2. In addition, the GFP-tagged herpes simplex virus 1 (HSV-1, KOS strain) and vesicular stomatitis virus (VSV, Indiana HR strain) were adopted in accordance with previously reported protocols and applications [12].

### 2.2. Cell Viability and Lactate Dehydrogenase Release Analysis

Cell viability was analyzed by using a cell counting kit-8 (CCK-8) assay kit. 3D4/21 cells were plated in 96-well culture plates. 3D4/21 cells were exposed to Mn^2+^ (0, 1, 10, 25, 50, 100, 200, 400 and 800 μM) for 12 h and 24 h respectively. Two hours prior to the termination of treatment, 10 µL of CCK-8 reagent was supplemented into the culture medium of each well. The optical density (OD) values were subsequently measured at a wavelength of 450 nm using a microplate reader.

Lactate dehydrogenase (LDH) is a cytoplasmic enzyme that is released extracellularly once the cell membrane is damaged or during cellular death. Accordingly, the cytotoxicity induced by Mn^2+^ was evaluated by detecting LDH release into the supernatant, utilizing a commercial LDH cytotoxicity assay kit (Beyotime, Shanghai, China) in strict accordance with the manufacturer’s standard protocols. The absorbance of each sample was ultimately determined at 490 nm via microplate spectrophotometry.

### 2.3. Preparation of Homozygous KO 3D4/21 Cell Clones by CRISPR-Cas9 Approach

CRISPR guide RNAs (gRNAs) specific to porcine STING and IRF3 genes were designed with an online tool provided by Benchling (www.benchling.com, accessed on 2025.01.10). Based on the predicted scores, two pairs of gRNAs were chosen for each target gene. The DNA fragments encoding these gRNAs were individually cloned into the BbsI restriction enzyme site of the pX458-EGFP vector, and the recombinant pX458-gRNA plasmids were verified by DNA sequencing to ensure correctness. 3D4/21 cells were seeded in 6-well plates at a density of 6–8 × 10^5^ cells per well, followed by co-transfection with each recombinant pX458-gRNA plasmid using Lipofectamine 2000 transfection reagent. 24 h post-transfection, GFP-positive cells were isolated via flow cytometry and then seeded into 96-well plates using the limiting dilution method to facilitate monoclonal cell growth. Western blotting was employed to detect the protein expression levels of STING or IRF3 in the obtained single 3D4/21 cell clones. Additionally, genomic DNA editing efficiency in all 3D4/21 cell clones was evaluated by PCR analysis. The detailed experimental protocol was performed as described in previous reports [13].

### 2.4. Transcriptomic Analysis

Transcriptomic analysis was performed by TsingkeBiotechnology (Beijing, China). In detail, the STING^−^/^−^ 3D4/21 cells were exposed to 0 and 75 μM of Mn^2+^ for 12 h, and then the RNA was extracted. For transcriptome library construction, 1 μg of total RNA was employed as the starting material for each individual sample. Sequencing libraries were generated using the NEBNext^®^ Ultra™ RNA Library Prep Kit for Illumina^®^ (NEB, Ipswich, MA, USA), with unique index labels assigned to all samples. Subsequently, clustering of index-tagged samples was performed on a cBot Cluster Generation System using the TruSeq PE Cluster Kit v3-cBot-HS (Illumia San Diego, CA, USA). Following the completion of cluster amplification, the constructed libraries were sequenced on the Illumina NovaSeq platform to generate 150 bp paired-end reads. Gene Ontology (GO) enrichment analysis was conducted to annotate differentially expressed genes (DEGs) into three primary functional categories: Biological Process, Cellular Component, and Molecular Function. Furthermore, all identified DEGs were mapped to the Kyoto Encyclopedia of Genes and Genomes (KEGG) database, enabling their classification into signaling cascades, metabolic networks, and disease-associated pathways. The detailed experimental protocol was performed as described in previous reports [14,15].

### 2.5. Western Blotting Analysis

After the cells were treated according to the experimental design, they were harvested via trypsin digestion. Total cellular proteins were extracted using RIPA lysis buffer. The concentration of the extracted total proteins was determined and adjusted utilizing a BCA protein assay kit. Subsequently, the total proteins were collected, mixed with loading buffer, and subjected to boiling for 10 min. The extracted proteins were loaded onto a sodium dodecyl sulfate-polyacrylamide gel, where they were separated based on their molecular weights. Following gel separation, the proteins were transferred onto polyvinylidene fluoride membranes. These membranes were first incubated in a 5% skim milk solution; afterward, they were probed with the specified primary antibodies and incubated overnight at 4 °C. After washing, the membranes were further incubated with secondary antibodies for 2 h at room temperature. Finally, the protein signals were detected using an ECL detection system.

The antibodies against phosphorylated-TBK1 mAb (p-TBK1, 5483S), TBK1 mAb (3504S), IRF3 mAb (11904S) and GFP mAb (2956) were acquired from Cell Signaling Technology (Boston, MA, USA). The rabbit p-IRF3 (Ser385) was purchased from Thermo Fisher (Sunnyvale, CA, USA). The antibodies against cleaved caspase 3 (25128-1-AP), β-actin (66009-1-Ig) and GAPDH (60004-1-Ig) were purchased from Proteintech Group (Wuhan, China). The detailed experimental protocol was performed as described in a previous report [16].

### 2.6. Quantitative Reverse Transcription Polymerase Chain Reaction (qRT-PCR)

Total RNA isolation was carried out using TRIpure reagent purchased from Aidlab (Beijing, China). First-strand cDNA synthesis was performed with the HiScript^®^ 1st Strand cDNA Synthesis Kit supplied by Vazyme (Nanjing, China). The expression levels of target genes were detected via quantitative real-time PCR (qPCR) using ChamQ Universal SYBR qPCR Master Mix (Vazyme, Nanjing, China), and the reactions were run on a StepOne Plus real-time PCR system (Applied Biosystems, Foster City, CA, USA). The qPCR protocol was set as follows: an initial denaturation step at 95 °C for 30 s, followed by 40 amplification cycles consisting of 95 °C for 5 s (denaturation) and 60 °C for 1 min (annealing and extension). β-actin was used as an internal reference gene to normalize the expression data. The relative mRNA abundance of target genes was calculated using the 2^−ΔΔCT^ method. The sequence of qPCR primers used in this study is listed in Table 1.

### 2.7. Cell Apoptosis Analysis

The proportion of apoptotic cells was evaluated using an Annexin V-fluorescein isothiocyanate (Annexin V-FITC) apoptosis detection kit (BD Biosciences, Franklin Lakes, NJ, USA). Briefly, cells were digested with EDTA-free trypsin and harvested into 1.5 mL centrifuge tubes. After being washed with binding buffer, the cells were resuspended in the same buffer. Subsequently, Annexin V-FITC and propidium iodide (PI) staining solutions were added sequentially. The cells were then incubated at room temperature for 15 min in the dark, and the stained cells were immediately detected using a flow cytometer (LSRFortessa, BD Biosciences, USA). The detailed experimental protocol was performed as described in a previous report [17].

### 2.8. Level of Intracellular Reactive Oxygen Species Analysis

The level of ROS was detected by using an ROS assay kit (S0033S, beyotime, Shanghai, China). After treatment, the cells were washed with PBS and resuspended in PBS with 10 µM DCFH. The cells were incubated at 37 °C for 40 min. Then, the cells were washed twice with PBS and resuspended in PBS to detect ROS accumulation using flow cytometry at a wavelength pair of 488/525 nm.

The intracellular ROS level was determined using an ROS assay kit (cat. no. S0033S, Beyotime Biotechnology, Shanghai, China). Following the designated treatment, cells were rinsed with PBS and then resuspended in PBS containing 10 µM 2′,7’-dichlorodihydrofluorescein diacetate (DCFH). The cells were incubated at 37 °C for 40 min, after which they were washed twice with PBS and resuspended in fresh PBS. ROS accumulation was subsequently detected by flow cytometry at an excitation wavelength of 488 nm and an emission wavelength of 525 nm.

### 2.9. Statistical Analysis

The results were analyzed by using SPSS (SPSS 20.0 for Windows) and presented as the mean ± standard deviation (SD). The differences between different groups were analyzed by using a one-way analysis of variance (ANOVA). *p* < 0.05 was considered statistically significant.

## 3. Results

### 3.1. Analysis of Cell Viability

**Figure 1:**
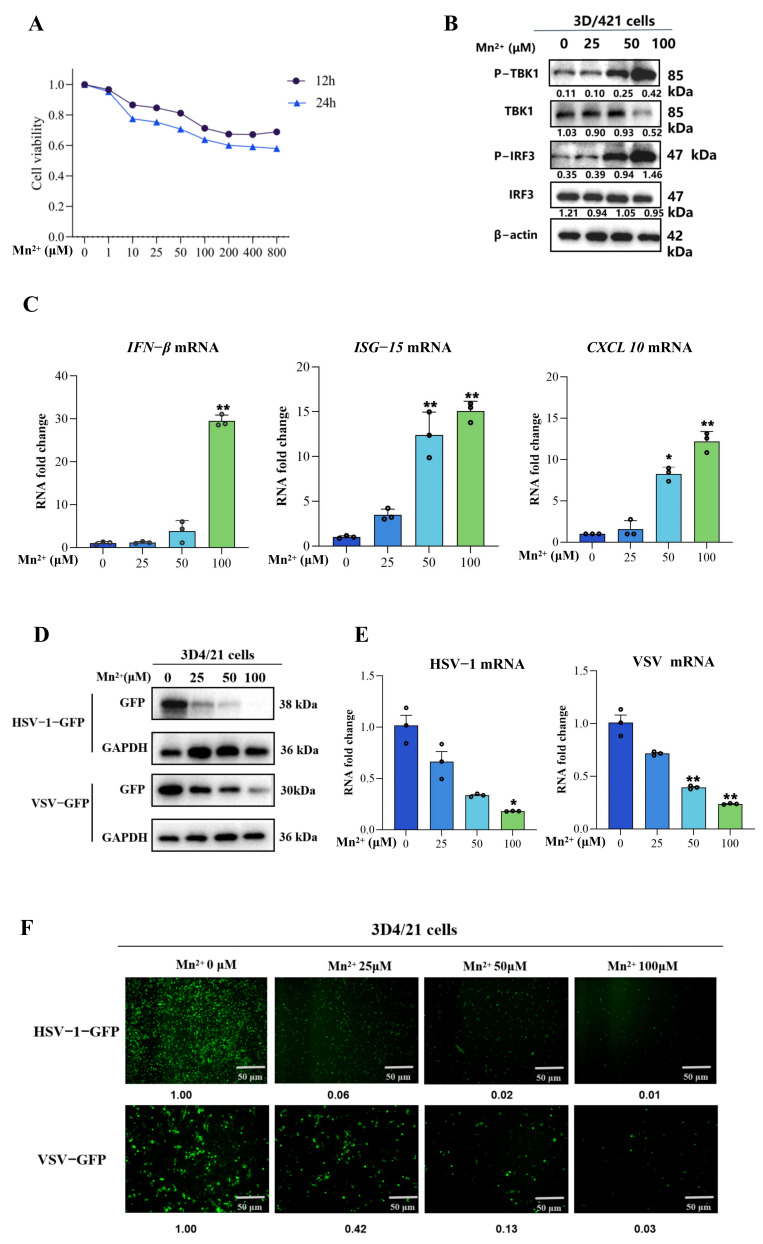
The effects of Mn^2+^ on the cell viability, IFN signaling and antiviral functions. (**A**) The impact of Mn^2+^ on the viability of 3D4/21 cells was determined using the CCK-8 assay. 3D4/21 cells were exposed to 0 μM, 1 μM, 10 μM, 25 μM, 50 μM, 100 μM, 200 μM, 400 μM and 800 μM for 12 h and 24 h. (**B**) The expressions of IFN signaling-related proteins were detected after treatment with Mn^2+^ in 3D4/21 cells. The expressions of p-TBK1, TBK1, p-IRF3 and IRF3 were normalized to β-actin, and densitometry values are given under the protein bands. (**C**) The expressions of the IFN signaling-related genes including IFN-β, ISG-15 and CXCL 10 were detected by using qPCR. (**D**–**F**) The 3D4/21 cells were pre-treated with various concentrations of Mn^2+^ (0 μM, 25 μM, 50 μM, and 100 μM) for 12 h, followed by infection with HSV-1-GFP (MOI 0.01) for 16 h, or VSV-GFP (MOI 0.001) for 12 h. (**D**) The replications of HSV-1-GFP and VSV-GFP were examined by using Western blotting. The expression of GFP in the WB results was normalized to GAPDH, with the densitometry values presented. (**E**) The replications of HSV-1-GFP and VSV-GFP were examined by using RT-PCR. (**F**) The replications of HSV-1-GFP and VSV-GFP were examined by using fluorescence microscopy. The fluorescence intensity of GFP was normalized to the Mn^2+^ (0 μM) group, and the relative fluorescence intensity values are presented below the fluorescence images. Note that all of the experiments were conducted with three biological replicates per sample group. Microscopy was performed using a 10× eyepiece combined with a 10× scope. The circles in the graphs represent individual biological replicates. Values represent the mean ± S.D. * *p*  <  0.05, ** *p*  <  0.01.

The effects of Mn^2+^ on the cell viability of 3D4/21 cells were detected by using the CCK8 assay. 3D4/21 cells were exposed to 0, 1, 10, 25, 50, 100, 200, 400 and 800 μM for 12 h and 24 h. As shown in Figure 1A, the viability of the 3D4/21 cells decreased in a dose-dependent manner. When the concentration of Mn^2+^ reached 100 μM, after 12 h of treatment, the viability of 3D4/21 cells was approximately 80%. Thus, in this study, we selected 25, 50 and 100 μM Mn^2+^ and 12 h as the treatment concentrations and time.

### 3.2. Mn^2+^ Could Promote IFN Signaling and Exert Antiviral Functions

To investigate the effects of Mn^2+^ on IFN signaling, the expressions of the IFN signaling-related proteins and genes were detected after treatment with Mn^2+^ in the 3D4/21 cells. As shown in Figure 1B,C, Mn^2+^ could increase the phosphorylation of TBK1 (p-TBK1) and IRF3 (p-IRF3) and promote the gene expressions of *IFN-β*, *ISG-15* and *CXCL 10* in a dose-dependent manner. Furthermore, to investigate the effect of Mn^2+^ on virus replication, the 3D4/21 cells were pre-treated with different concentrations of Mn^2+^ (0, 25, 50, and 100 μM) for 12 h, followed by infection with HSV-1-GFP (MOI 0.01) for 16 h, or VSV-GFP (MOI 0.001) for 12 h. As shown in Figure 1D–F, the replications of HSV-1-GFP and VSV-GFP were examined by using Western blotting, RT-PCR and fluorescence microscopy. The results revealed that Mn^2+^ could suppress the replication of HSV-1 and VSV in a dose-dependent manner. These results suggest that Mn^2+^ could promote IFN signaling and exert antiviral functions against HSV-1-GFP and VSV-GFP.

### 3.3. cGAS-STING Pathway Is Essential for Mn^2+^ to Promote IFN Signaling, but Is Not Essential for Triggering Antiviral Functions

**Figure 2:**
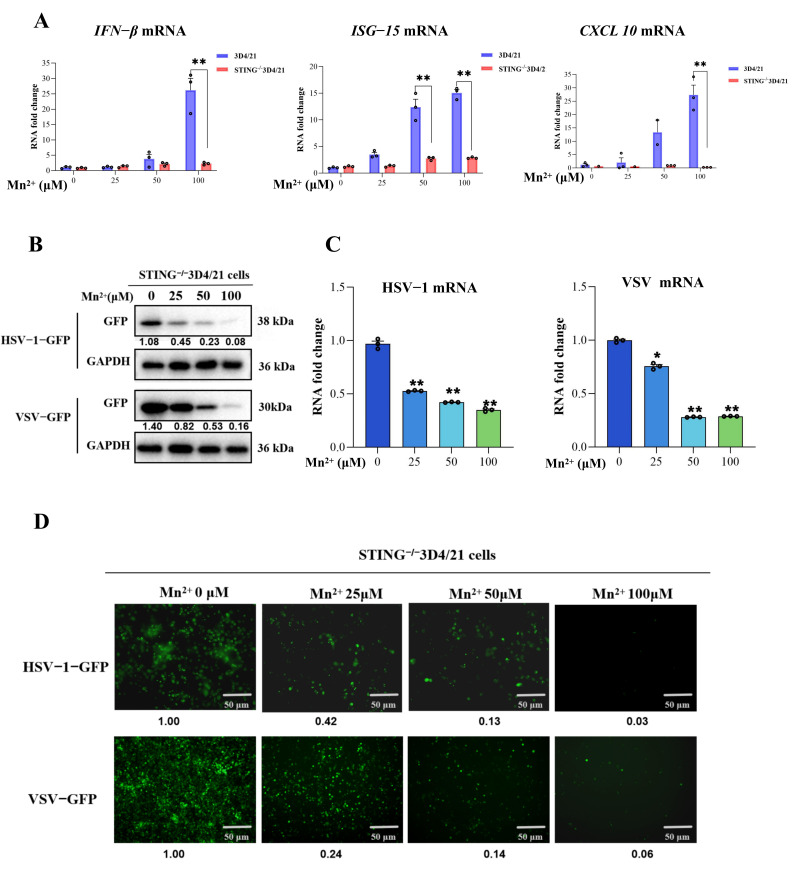
The role of the cGAS-STING pathway in Mn^2+^ promoting IFN signaling and exerting antiviral functions. (**A**) The mRNA expression levels of IFN signaling-related genes including IFN-β, ISG-15, and CXCL10 were detected by using RT-PCR. WT3D4/21 and STING^−/−^ 3D4/21 cells were treated with different concentrations (0 μM, 25 μM, 50 μM, and 100 μM) of Mn^2+^ for 12 h, respectively. (**B**–**D**) The role of the cGAS-STING pathway in Mn^2+^ exerting antiviral functions was examined by Western blotting. STING^−/−^ 3D4/21 cells were pre-treated with various concentrations of Mn^2+^ (0 μM, 25 μM, 50 μM, and 100 μM) for 12 h, followed by infection with HSV-1-GFP (MOI 0.01) for 16 h, or VSV-GFP (MOI 0.001) for 12 h. (**B**) The replication of HSV-1-GFP and VSV-GFP was examined by Western blotting. The expression of GFP in the WB results was normalized to GAPDH; the densitometry values are presented under the protein bands. (**C**) The replication of HSV-1-GFP and VSV-GFP was examined by RT-PCR. (**D**) The replication of HSV-1-GFP and VSV-GFP was examined by fluorescence microscopy. The fluorescence intensity of GFP was normalized to the Mn^2+^ (0 μM) group, and the relative fluorescence intensity values were presented below the fluorescence images. Note that all of the experiments were conducted with three biological replicates per sample group. Microscopy was performed using a 10× eyepiece combined with a 10× scope. The circles in the graphs represent individual biological replicates. Values represent the mean  ±  S.D. * *p*  <  0.05, ** *p*  <  0.01.

Mn^2+^ has been found to promote the sensitivity of the cGAS-STING pathway for double-stranded DNA. Thus, the role of the cGAS-STING pathway in Mn^2+^ promoting IFN signaling and exerting antiviral functions was detected by using STING^−^^/−^ 3D4/21. WT 3D4/21 and STING^−/−^ 3D4/21 cells were treated with different concentrations (0, 25, 50, and 100 μM) of Mn^2+^ for 12 h. Then, the mRNA expression levels of IFN signaling-related genes including IFN-β, ISG-15, and CXCL10 were detected by using RT-PCR. The results showed (Figure 2A) that Mn^2+^ could influence the mRNA expressions of IFN-β, ISG-15, and CXCL10 in WT 3D4/21, while the mRNA expressions of IFN-β, ISG-15, and CXCL10 were not significantly changed in STING^−^/^−^ 3D4/21 cells after treatment with different concentrations of Mn^2+^.

Furthermore, to investigate the effect of Mn^2+^ on virus replication in the STING^−^/^−^3D4/21 cells, the STING^−^/^−^3D4/21 cells were pre-treated with different concentrations (0, 25, 50, and 100 μM) of Mn^2+^ for 12 h, followed by infection with HSV-1-GFP (MOI 0.01) for 16 h or VSV-GFP (MOI 0.001) for 12 h. The replication of HSV-1-GFP and VSV-GFP was examined by Western blotting, RT-PCR and fluorescence microscopy. The results revealed that Mn^2+^ still retains its antiviral activity against HSV-1 and VSV after knocking out the STING gene in 3D4/21 cells.

Collectively, these results indicate that the cGAS-STING pathway is essential for Mn^2+^ promotion of IFN signaling, but it is not essential for Mn^2+^ triggering of antiviral functions. Mn^2+^ could exert antiviral functions independent of the cGAS-STING-IFN pathway.

### 3.4. Transcriptomic Analysis of the Effects of Mn^2+^ on STING^−^/^−^ 3D4/21 Cells

**Figure 3:**
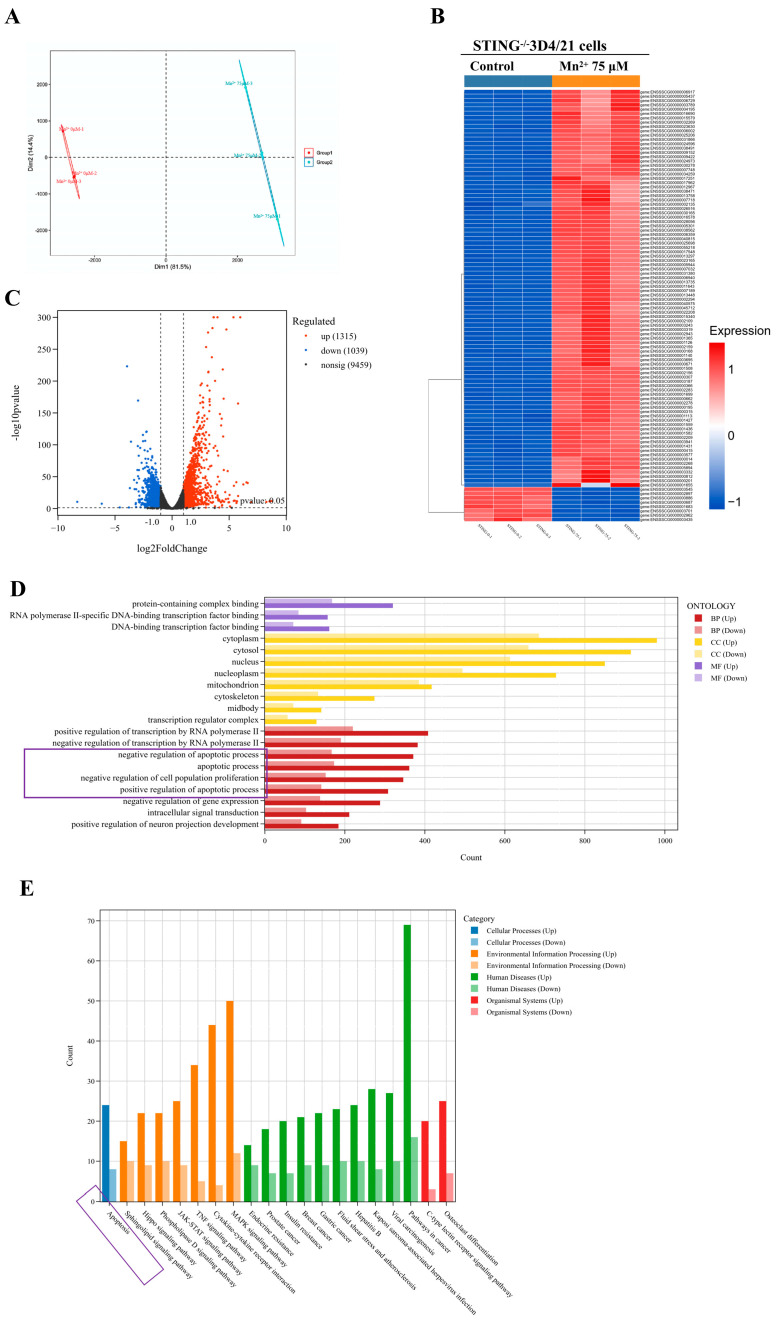
The transcriptomic analysis of the effects of Mn^2+^ on STING^−^/^−^ 3D4/21 cells. The STING^−^/^−^ 3D4/21 cells were exposed to 75 μM of Mn^2+^ for 12 h. A total amount of 1 μg RNA per sample was used for RNA sample preparations. (**A**) The inter-group difference and intra-group difference were analyzed by using principal component analysis. (**B**) A heatmap plot of the DEGs across all samples. (**C**) The distribution of upregulated and downregulated DEGs analyzed by volcano maps. (**D**) The functional classification of the DEGs was analyzed by GO enrichment analysis. (**E**) The biological pathways of the DEGs were analyzed by using KEGG.

In order to explore the antiviral mechanism of Mn^2+^ independent of the cGAS-STING-IFN signaling pathway, the transcriptomic profiles of STING^−^/^−^ 3D4/21 cells were determined after treatment with 75 μM Mn^2+^ for 12 h. Three independent biological replicates (*n* = 3) were adopted for each group. The inter-group differences and intra-group differences were analyzed using principal component analysis. The results revealed a pronounced divergence in transcriptomic profiles between groups, whereas minimal variation was observed within each group, indicative of the high reproducibility of the experimental treatments (Figure 3A). The heatmap of clustered DEGs showed that two groups had obviously distinct transcriptomic profiles compared to each other (Figure 3B). The distribution of upregulated and downregulated DEGs was analyzed by volcano maps (Figure 3C). In comparison with the control group, there were a total of 2624 DEGs in the Mn^2+^-treated group. Among these DEGs, 1315 genes were upregulated and 1309 genes were downregulated. To reveal the biological functions of the DEGs between the Mn^2+^-treated group vs the control group, Gene Ontology (GO) and Kyoto Encyclopedia of Genes and Genomes (KEGG) enrichment analyses were performed. The functional classification of the DEGs was analyzed by GO enrichment analysis. GO analysis showed (Figure 3D) that there are significantly enriched gene sets in the categories of biological process (BP), cellular component (CC) and molecular function (MF), respectively. The top three most significant changes in biological process are transcription by RNA negative polymerase regulation II, apoptotic process, and cell proliferation. Furthermore, the biological pathways of the DEGs were analyzed by using KEGG analysis. The KEGG analysis revealed (Figure 3E) that the DEGs were significantly enriched in several key pathways (*p* < 0.05), including apoptosis, the JAK-STAT signaling pathway, the TNF signaling pathway and the MAPK signaling pathway. Collectively, both the GO and KEGG analyses suggested that treatment with Mn^2+^ could induce a significant change in the apoptotic process in STING^−^/^−^ 3D4/21 cells.

### 3.5. Mn^2+^ Could Induce Cell Apoptosis in WT 3D4/21 and STING^−^/^−^ 3D4/21 Cells

**Figure 4:**
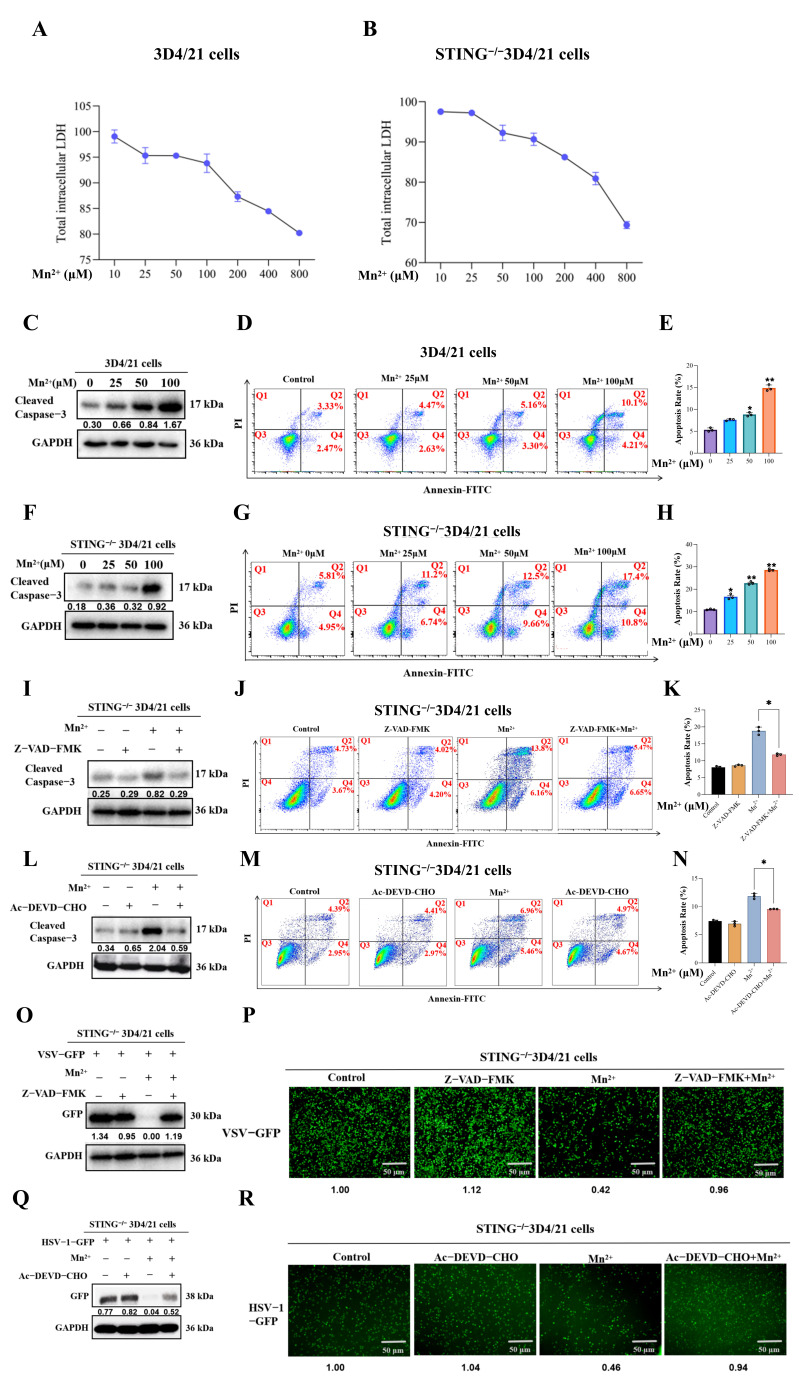
The role of cell apoptosis in the antiviral effect of Mn^2+^. (**A**,**B**) Mn^2+^-induced cytotoxicity was measured by monitoring LDH release into the culture media by LDH cytotoxicity assay kit. (**C**–**E**) The effects of Mn^2+^ on the cell apoptosis of WT 3D4/21 cells were investigated. (**F**–**H**) The effects of Mn^2+^ on the cell apoptosis of STING^−/−^ 3D4/21 cells were investigated. (**I**–**K**) The apoptosis inhibitor Z-VAD-FMK can effectively suppress Mn^2+^-induced apoptosis in STING^−^/^−^ 3D4/21 cells. (**L**–**N**) The apoptosis inhibitors Ac-DEVD-CHO can effectively suppress Mn^2+^-induced apoptosis in STING^−^/^−^ 3D4/21 cells. (**O**,**P**) Apoptosis is involved in the anti-HSV-1-GFP virus effect of Mn^2+^ in STING^−/−^ 3D4/21 cells. (**Q**,**R**) Apoptosis is involved in the anti-VSV-GFP virus effect of Mn^2+^ in STING^−/−^ 3D4/21 cells. Note the expression of cleaved caspase 3 and GFP in the WB results is normalized to GAPDH; the densitometry values are presented under the protein bands. The fluorescence intensity of GFP was normalized to the Mn^2+^ (0 μM) group, and the relative fluorescence intensity values were presented below the fluorescence images. All of the experiments were conducted with three biological replicates per sample group. Microscopy was performed using a 10× eyepiece combined with a 10× scope. The circles in the graphs represent individual biological replicates. Values represent the mean  ±  S.D. * *p*  <  0.05, ** *p*  <  0.01.

The results from transcriptomic analysis show that numerous DGEs in STING^−/−^ 3D4/21 cells treated with Mn^2+^ for 12 h were enriched in the apoptotic process. Thus, the effects of Mn^2+^ on cell apoptosis in WT and STING^−/−^ 3D4/21 cells were investigated. As shown in Figure 4A,B, compared with the control group, after treatment with different concentrations (0, 1, 10, 25, 50, 100, 200, 400, and 800 μM) of Mn^2+^ for 12 h, the LDH release rates were increased in a dose-dependent manner in both WT and STING^−/−^ 3D4/21 cells. Results from Western blotting showed (Figure 4C,F) that, compared with the control group, Mn^2+^ treatment increased the expression level of cleaved caspase 3 in a dose-dependent manner in WT and STING^−^/^−^ 3D4/21 cells. Similarly, results from flow cytometry (Figure 4D,E,G,H) indicated that Mn^2+^ treatment induced a dose-dependent increase in apoptotic ratios in both WT and STING^−^/^−^ 3D4/21 cells. Collectively, these results indicate that Mn^2+^ could induce cell apoptosis in WT and STING^−^/^−^ 3D4/21 cells in a dose-dependent manner.

### 3.6. Apoptosis Is Involved in the Antiviral Effect of Mn^2+^ in STING^−/−^ 3D4/21 Cells

Apoptotic signaling is an important pathway in the host response to viral infection. Thus, the role of the apoptotic pathway in Mn^2+^-mediated antiviral functions was detected by using the apoptotic inhibitor. The STING^−^/^−^3D4/21 cells were pre-treated with Z-VAD-FMK (pan-caspase inhibitor) and Ac-DEVD-CHO (caspase-3-specific inhibitor) for 30 min, followed by co-treatment with 50 μM Mn^2+^ for 12 h. The results from Western blotting and flow cytometry (Figure 4I–N) have indicated that both the apoptosis inhibitors Z-VAD-FMK and Ac-DEVD-CHO can effectively suppress Mn^2+^-induced apoptosis in STING^−^/^−^ 3D4/21 cells.

Furthermore, the role of apoptosis on Mn^2+^ against HSV-1-GFP and VSV-GFP was detected. The expression of the viral reporter GFP was detected by Western blotting, and the results showed (Figure 4O,Q) that compared with the Mn^2+^-alone treatment group, the GFP expression of HSV-1-GFP and VSV-GFP significantly increased in the co-treated-with-Mn^2+^-and-apoptotic-inhibitors groups. The changes in GFP fluorescence were observed by using fluorescence microscopy (Figure 4P,R), and the GFP fluorescence of HSV-1-GFP and VSV-GFP was significantly elevated in the groups co-treated with Mn^2+^ and apoptotic inhibitors. These results indicated that inhibiting the apoptotic signal could suppress Mn^2+^-mediated antiviral activity in STING^−^/^−^ 3D4/21 cells.

### 3.7. Oxidative Stress Is Involved in Mn^2+^-Induced Cell Apoptosis and Antiviral Effects

**Figure 5:**
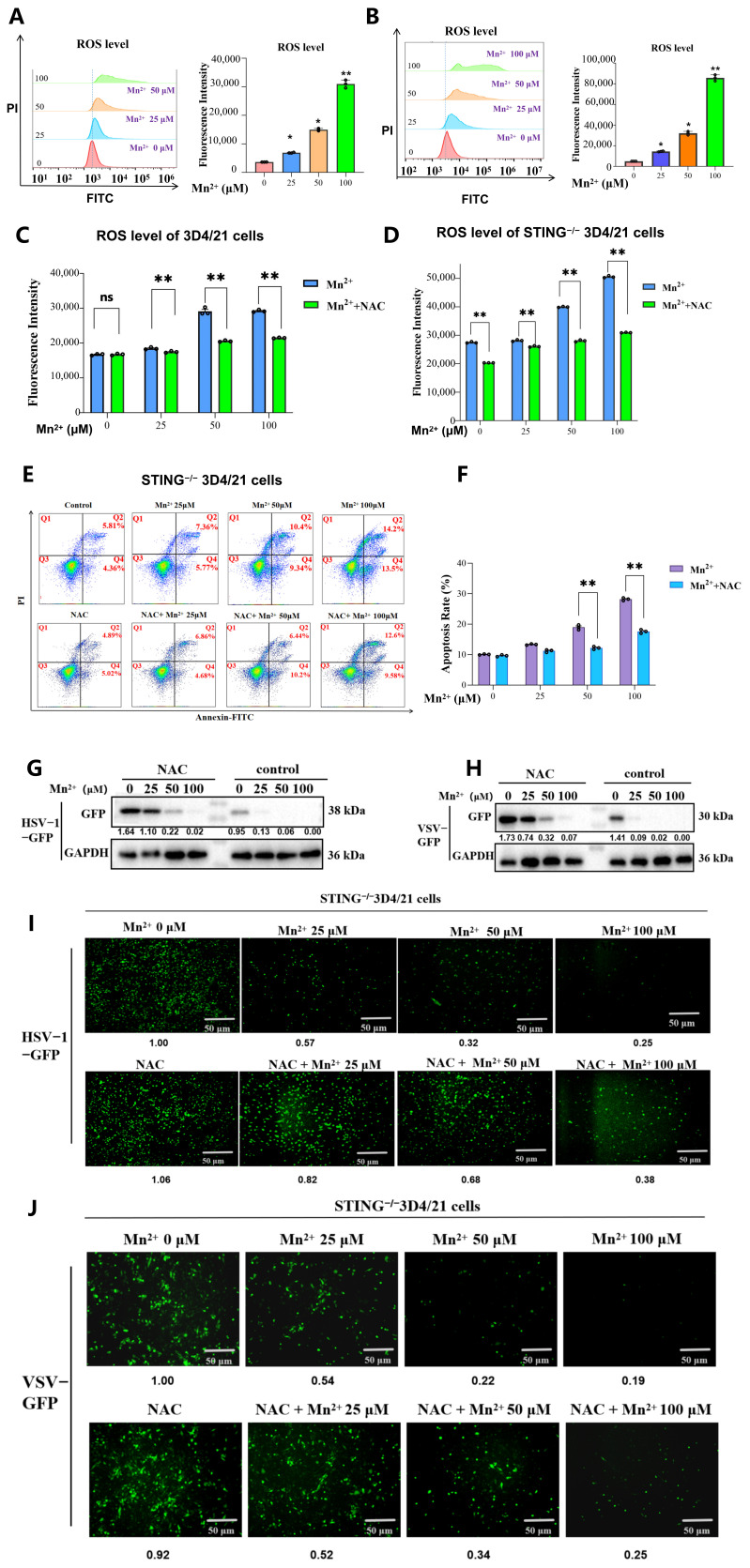
Mn^2+^ exerts antiviral activity through the ROS-apoptosis signaling pathway. (**A**,**B**) The effects of Mn^2+^ on ROS level in WT and STING^−/−^ 3D4/21 cells were detected by using flow cytometry. WT and STING^−/−^ 3D4/21 cells were treated with different concentrations (0, 25, 50, and 100 μM) of Mn^2+^ for 12 h. (**C**,**D**) The role of antioxidant NAC in the production of ROS induced by Mn^2+^ was analyzed by using flow cytometry. Cells were pre-treated with 10 mmol/L NAC for 30 min, followed by treatment with different concentrations (0, 25, 50, and 100 μM) of Mn^2+^ in WT and STING^−/−^ 3D4/21 cells. (**E**,**F**) The role of antioxidant NAC in Mn^2+^-induced apoptosis was analyzed by using flow cytometry. (**G**–**J**) The role of oxidative stress on Mn^2+^ activity against HSV-1-GFP and VSV-GFP was detected. (**G**,**H**) The expression of the viral reporter GFP was detected by Western blotting. (**I**,**J**) Changes in GFP fluorescence were observed by using fluorescence microscopy. Note that the expression of cleaved caspase 3 and GFP in the WB results was normalized to GAPDH; the densitometry values are presented under the protein bands. The fluorescence intensity of GFP was normalized to the Mn^2+^ (0 μM) group, and the relative fluorescence intensity values are presented below the fluorescence images. All of the experiments were conducted with three biological replicates per sample group. Microscopy was performed using a 10× eyepiece combined with a 10× objective. The circles in the graphs represent individual biological replicates. Values represent the mean  ±  S.D. * *p*  <  0.05, ** *p*  <  0.01; ns indicates no significant difference .

In order to investigate the mechanisms of Mn^2+^-induced apoptosis, the effect of Mn^2+^ on cellular oxidative stress was detected. The intracellular ROS level was quantified by flow cytometry by using DCFH-DA staining. WT and STING^−^/^−^ 3D4/21 cells were treated with different concentrations (0, 25, 50, and 100 μM) of Mn^2+^ for 12 h. Comparing with the control group, the intracellular ROS levels in WT and STING^−^/^−^3D4/21 cells were significantly increased in the groups treated with Mn^2+^ in a dose-dependent manner (Figure 5A,B).

Furthermore, in order to investigate the role of ROS in Mn^2+^-induced apoptosis, the antioxidant N-acetylcysteine (NAC) was selected to inhibit the production of ROS. WT and STING^−^/^−^ 3D4/21 cells were pre-treated with 10 mmol/L NAC for 30 min, followed by treatment with different concentrations (0, 25, 50, and 100 μM) of Mn^2+^ for 12 h. The results showed that compared with the Mn^2+^ alone treatment group, the intracellular ROS levels in the groups co-treated with NAC and Mn^2+^ were significantly decreased in both 3D4/21 and STING^−^/^−^ 3D4/21 cells (Figure 5C,D). The results from flow cytometry indicated that compared with the Mn^2+^ alone treatment group, the apoptotic levels in the groups co-treated with NAC and Mn^2+^ significantly decreased in both 3D4/21 and STING^−^/^−^ 3D4/21 cells (Figure 5E,F).

Additionally, the role of oxidative stress in Mn^2+^ activity against HSV-1-GFP and VSV-GFP was detected. The expression of the viral reporter GFP was detected by Western blotting, and changes in GFP fluorescence were observed using fluorescence microscopy. Results from Western blotting showed (Figure 5G,H) that, compared with the Mn^2+^-alone treatment group, the GFP expression of HSV-1-GFP and VSV-GFP significantly increased in the group co-treated with NAC and Mn^2+^. Similarly, the GFP fluorescence of HSV-1-GFP and VSV-GFP was significantly elevated in the group co-treated with NAC and Mn^2+^ (Figure 5I,J). Collectively, these results indicate that Mn^2+^ could exert antiviral activity through the ROS-apoptosis signaling pathway.

### 3.8. When Both IFN and Apoptotic Signals Are Absent, Mn^2+^ Loses Its Antiviral Activities

**Figure 6:**
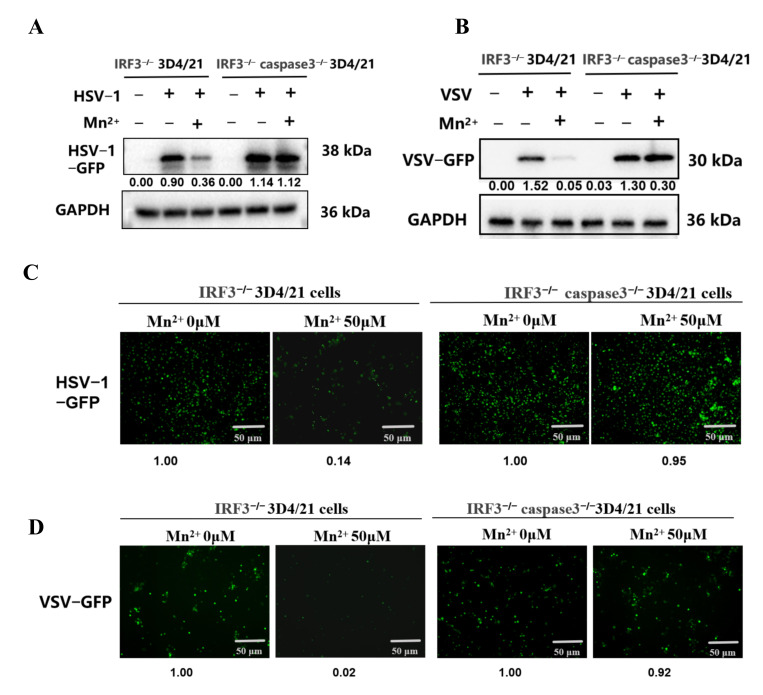
Blocking IFN and apoptotic signaling eliminates the antiviral effects of Mn^2+^. The IRF3^−/−^ 3D4/21 cells and IRF3^−/−^ caspase3^−/−^ 3D4/21 cells were pre-treated with 50 μM Mn^2+^ for 12 h, followed by infection with HSV-1-GFP (MOI 0.01) for 16 h, or VSV-GFP (MOI 0.001) for 12 h. (**A**,**B**) The expression of the viral reporter GFP was detected by Western blotting. (**C**,**D**) The changes in GFP fluorescence were observed by using fluorescence microscopy. Note that the expression of cleaved caspase 3 and GFP in the WB results was normalized to GAPDH; the densitometry values are presented under the protein bands. The fluorescence intensity of GFP was normalized to the Mn^2+^ (0 μM) group, and the relative fluorescence intensity values are presented below the fluorescence images. All of the experiments were conducted with three biological replicates per sample group. Microscopy was performed using a 10× eyepiece combined with a 10× scope.

The above data suggest that Mn^2+^ can exert antiviral activity through the cGAS-STING-IFN and apoptosis signaling pathways. To detect whether there are other pathways involved in the antiviral effect of Mn^2+^ besides these two pathways, 3D4/21 cells with double gene knockout of IRF3 and caspase3 were prepared. The IRF3 gene was knocked out to block the cGAS-STING signaling pathway, and caspase3 was knocked out to inhibit the apoptotic pathway. The results from Western blotting showed (Figure 6A,C) that for the single knockout of IRF3 cells (IRF3^−/−^ 3D4/21 cells), treatment with 50 μM Mn^2+^ could inhibit the GFP protein expressions of HSV1-GFP and VSV-GFP, while in the dual knockout of IRF3 and caspase3 cells (IRF3^−/−^ caspase3^−/−^ 3D4/21 cells), treatment with 50 μM Mn^2+^ could not inhibit the GFP protein expressions of HSV1-GFP and VSV-GFP. Similarly, the results from fluorescence microscopy also showed (Figure 6B,D) that the GFP fluorescence of HSV-1-GFP and VSV-GFP was significantly decreased in the IRF3^−/−^ 3D4/21 cells after treatment with 50 μM Mn^2+^, while the GFP fluorescence did not change significantly in the IRF3^−/−^ caspase3^−/−^ 3D4/21 cells treated with 50 μM Mn^2+^. These results indicate that single knockout of IRF3, which leads to IFN signaling deficiency, does not abrogate the antiviral activity of Mn^2+^. In contrast, dual knockout of IRF3 and caspase3, resulting in concurrent loss of IFN and apoptotic signaling, eliminates the antiviral effects of Mn^2+^.

## 4. Discussion

Apoptosis represents a non-inflammatory programmed cell death process, which displays typical morphological changes including cell shrinkage, nuclear condensation, and plasma membrane blebbing [18,19]. As a fundamental process in multicellular organisms, apoptosis is essential for preserving tissue homeostasis and eliminating redundant cells. This form of programmed cell death is governed by several signaling cascades, mainly the intrinsic (mitochondrial) pathway and the extrinsic (death receptor) pathway [20]. The intrinsic apoptotic pathway is initiated by multiple intracellular stress signals, such as hypoxia, DNA damage and oxidative stress. This pathway is precisely regulated by the B-cell Lymphoma 2 (Bcl-2) protein family, which controls the permeability of the outer mitochondrial membrane and the release of apoptogenic molecules, including cytochrome c [21]. Following activation, a cascade of biochemical reactions occurs, such as caspase activation, DNA fragmentation and the degradation of cellular components. The extrinsic apoptotic pathway is triggered by ligands such as tumor necrosis factor (TNF), which promotes the assembly of the death-inducing signaling complex (DISC) and subsequently activates caspase-8 and its downstream effector caspases [22].

Apoptosis is a critical cellular process that plays a pivotal role in maintaining tissue homeostasis and defending against viral infections. The pathogenesis of viral infections involves dynamic interactions between viruses and hosts, which can result in different outcomes including cell death, elimination of the virus or latent infection. The intricate relationship between apoptosis and viral infections is multifaceted, with apoptosis acting as a double-edged sword. It not only constitutes a primary defense strategy for the host to restrict viral replication and spread but can also be hijacked or modulated by viruses to facilitate their survival and pathogenicity [23]. Upon viral entry and replication, host cells initiate apoptotic signaling to eliminate infected cells before the virus completes its life cycle, thereby limiting viral progeny production and intercellular transmission [24]. In the current study, both GO and KEGG analyses suggested that treatment with Mn^2+^ could induce a significant change in the apoptotic process in STING^−^/^−^ 3D4/21 cells. This study has verified that Mn^2+^ can induce cell apoptosis in WT and STING^−^/^−^ 3D4/21 cells, and the apoptotic signaling pathway is involved in the antiviral activity of Mn^2+^.

ROS are highly reactive oxygen-containing molecules generated via partial reduction in molecular oxygen or other metabolic processes [25]. These species include oxygen-derived free radicals such as superoxide anions, hydroxyl radicals, and hydroperoxyl radicals, as well as singlet oxygen and reactive nitrogen radicals [26]. Under physiological conditions, ROS play indispensable roles in regulating cell cycle progression, proliferation, differentiation, migration, and apoptosis [27,28]. Excessive free radical generation can be induced by various factors, including ultraviolet radiation, prolonged stress, strenuous exercise, unhealthy diet, and stimulant abuse [29]. Abnormal ROS accumulation disturbs intracellular redox balance, thereby triggering oxidative stress and subsequent oxidative damage to cellular components [30,31]. However, it has been suggested that increasing ROS accumulation can induce substantial damage to proteins, nucleic acids, lipids, membranes, and organelles, thereby initiating cell death pathways such as apoptosis [32]. Mn^2+^ acts as an essential constituent of superoxide dismutase, which plays a major role in scavenging ROS during mitochondrial oxidative stress. Additionally, both manganese deficiency and manganese intoxication are linked to detrimental metabolic disorders and neuropsychiatric impairments.

Apoptosis comprises three distinct stages—induction, effector, and execution—each of which is significantly regulated by ROS and oxidative stress. The results from this study suggest that Mn^2+^ could induce the production of ROS, and ROS is involved in Mn^2+^-induced cell apoptosis and antiviral effects. It was indicated that Mn^2+^ could exert antiviral effects through the cGAS-STING-IFN signaling pathway. However, after knocking out the STING or IRF3 genes, Mn^2+^ still retains antiviral effects. This indicates that, beyond its role in the cGAS-STING-IFN axis, Mn^2+^ can also exert antiviral effects through other pathways. Our results indicate that Mn^2+^ can exert antiviral activity through the ROS-apoptosis signaling pathway in STING^−/−^3D4/21 cells. Interestingly, dual knockout of IRF3 and caspase 3, resulting in concurrent loss of IFN and apoptotic signaling, could eliminate the antiviral effects of Mn^2+^.

## 5. Conclusions

Mn^2+^ could exert antiviral effects not only through the cGAS-STING-IFN pathway but also via the ROS-apoptosis pathway.

## Figures and Tables

**Table 1:** Primers used for qPCR in this study.

Primers	Sequences (5′-3′)
HSV1 gB gene-F	5′-TTCTGCAGCTCGCACCAC-3′
HSV1 gB gene-R	5′-GGAGCGCATCAAGACCACC-3′
VSV glycoprotein gene-F	5′-TGCAAGGAAAGCATTGAACAA-3′
VSV glycoprotein gene-R	5′-GAGGAGTCACCTGGACAATCACT-3’
Porcine IFN-β-F	5’-TGAGCATTCTGCAGTACCTGA-3’
Porcine IFN-β-R	5’-CCGGAGGTAATCTGTAAGTCTGT-3’
Porcine ISG15-F	5’-ATCCTGGTGAGGAACGACAA-3’
Porcine ISG15-R	5’-GAAAGTCAGCCAGAACTGGTC-3’
Porcine ISG56-F	5’-ATGGGAGTTGGTCATTCAAGA-3’
Porcine ISG56-R	5’-CAGGTGTTTCACATAGGCCA-3’
Porcine ISG60 F	5’-CCAGACAACCCAGAATTCTCCT-3’
PorcineISG60 R	5’-AGAGCGCTGATGAAGTTGTTGC-3’
Porcine IL-8-F	5’-GTTTTTGAAGAGGGCTGAGAATTC-3’
Porcine IL-8-R	5’-CATGAAGTGTTGAAGTAGATTTGCTTG-3’
Porcine β-actin-F	5’-ATGAAGATCAAGATCATCGCG-3’
Porcine β-actin-R	5’-TCGTACTCCTGCTTGCTGATC-3’

## Data Availability

The original contributions presented in this study are included in the article. Further inquiries can be directed to the corresponding author.
